# Ethnobotanical Study of Medicinal Shrubs and Herbs Used by Forest-Fringe Communities of Ghana

**DOI:** 10.1155/sci5/1362301

**Published:** 2025-05-19

**Authors:** Michael Asigbaase, Linda Anaba, Daniel Adusu, Simon Abugre, Adisa Ayeley Musah, Collins Ayine Nsor, Daniel Akoto Sarfo

**Affiliations:** ^1^Department of Forest Sciences, University of Energy and Natural Resources, Sunyani, Ghana; ^2^Department of Theoretical and Applied Biology, College of Science, Kwame Nkrumah University of Science and Technology, Kumasi, Ghana; ^3^Department of Environmental Management, University of Energy and Natural Resources, Sunyani, Ghana; ^4^Department of Biological Sciences, University of Energy and Natural Resources, Sunyani, Ghana; ^5^Department of Forest Resources Technology, Faculty of Renewable Natural Resources, Kwame Nkrumah University of Science and Technology, Kumasi, Ghana

**Keywords:** apparency hypothesis, ethnobotanical survey, indigenous knowledge, medicinal plants, species use values, versatility hypothesis

## Abstract

Documenting and quantitatively assessing medicinal plants and indigenous knowledge in different social contexts is critical in providing nature-based solutions to contemporary issues. Therefore, our study quantitatively evaluated medicinal shrubs and herbs in forest-fringe communities of Ghana and tested the versatility, ecological apparency and sociodemographic traits and knowledge dynamics hypotheses. Structured questionnaires, interviews and field visits were used to conduct an ethnobotanical survey involving 78 respondents, selected based on random and snowball sampling techniques. The data were analysed using quantitative indices such as indigenous knowledge index (IKI), species use value (SUV), informant agreement ratio (IAR) and ethnobotanical importance value (EIV). To test the versatility and sociodemographic traits and knowledge dynamics hypotheses, linear mixed-effects regressions were conducted, while one-way ANOVA was used to test the ecological apparency hypothesis. The Jaccard dissimilarity index was used to assess the degree of uniqueness of diseases treated by plants. We found 69 medicinal shrubs and herbs, belonging to 35 plant families, used to treat 101 diseases in the study area. *Paullinia pinnata* L. recorded the highest SUV (18.2) values, whereas *Momordica charantia* L. recorded the highest EIV (22.326) values. We found support for the versatility and sociodemographic traits and knowledge dynamics hypotheses, but no support for the ecological apparency hypothesis. The IAR of the 16 disease categories evaluated in this study ranged from 0.50 to 0.77. The Jaccard index showed that diseases treated by using introduced or native plants were 65.6% dissimilar. Our findings have implications for the achievement of SDG 3 (good health and well-being). We concluded that sociodemographic traits influence ethnobotanical knowledge dynamics, while plants with multiple useful parts are the most versatile and recommend the conservation of biodiversity to enhance diversity of treatment options.

## 1. Introduction

Medicinal plants and other plant-based compounds have historically played a significant role in human health and well-being globally [1, 2]. Medicinal plants were the primary approach to treat various ailments before the inception of Western medicine. They are also widely recognized as a substantial global driver of significant evolutions in the pharmaceutical industry [3, 4]. It is estimated that about half of the global plant species are used in traditional medicine in different parts of the world [5]. These plants are primarily harvested from the wild and used by indigenous communities to cure various human ailments [5, 6]. Despite their ancient nature, many individuals around the global north and south still recognize traditional medicines as better alternatives as they are perceived to have minimal or no side effects compared to conventional medicines [7].

Despite historical advancements in pharmacology, many studies have estimated that about 80% of the world's population still relies on medicinal plants for healing many complex ailments [2, 8, 9]. The situation is more pronounced in developing countries, characterized by inadequate access to health services, ease of access to medicinal plants and cultural preferences for such remedies [6, 8]. Also, medicinal plants are an essential source of foreign exchange and revenue generation for many developing countries as they are well placed in global markets [10]. According to Sofowora et al. [11], the global market value of medicinal plant products exceeds $100 billion per annum. In addition, millions of people in developing countries derive their income, alternative livelihood support and employment opportunities from wild plant products [7, 12].

Like other developing regions of the world, many indigenous communities in Africa depend on medicinal plants for their primary health care [6, 12]. While this is primarily based on cultural and religious beliefs and a firm trust in this method, socioeconomic conditions such as limited health facilities, poverty and accessibility make medicinal plants more prevalent in this region [8]. In addition, knowledge and skills in using traditional medicine for preventive and curative care are deeply rooted in African culture [12]. They are transmitted from one generation to the other by tribal leaders.

In Ghana, a country in sub-Saharan Africa with rich floristic and ethnic diversity, herbal remedies are essential to the healthcare system, especially in rural areas [1, 3]. Having realized the critical role of traditional medicine in rural communities' social life, the government of Ghana established the Centre for Plant Medicine Research (CPMR) to conduct and promote various scientific activities that would improve herbal medicines [3]. While the actions of CPMR increased documentation on the usage of herbal medicines in Ghana, the diversity of plant species, cultural variations in the use of remedies and the widely oral transmission of indigenous knowledge on medicinal species threaten the sustainability of such approaches. In addition to the globalization trends, increased use of synthetic products and rapid land degradation put traditional knowledge of medicinal plants at risk of extinction [3, 7]. There is, therefore, an urgent need to document and conserve such knowledge, especially in local communities with a high risk of losing this practice.

**Figure 1:**
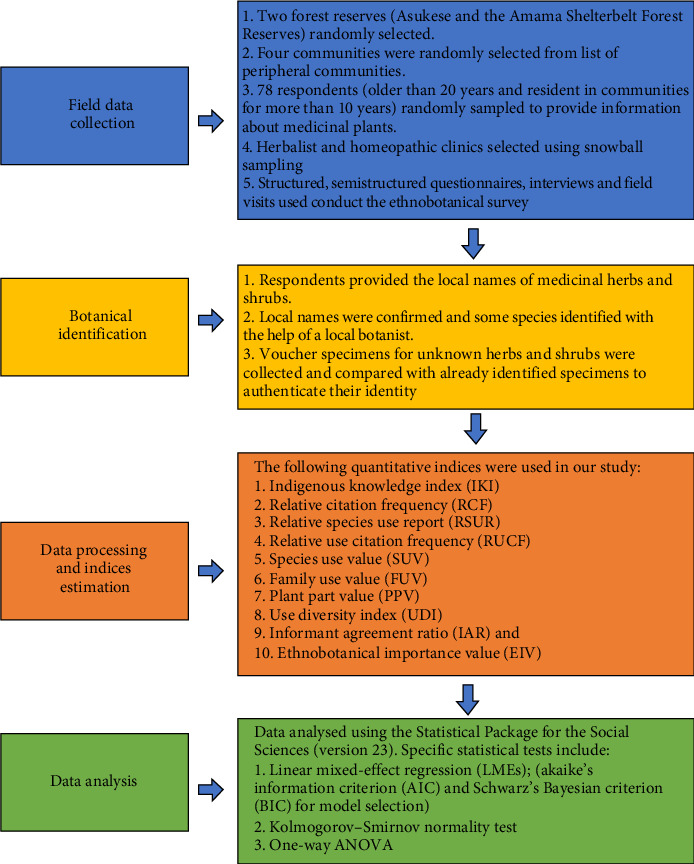
Research framework and process.

In recent decades of increased use and commercialization of medicinal plants globally, research on medicinal plants has gained significant attention among policymakers and the scientific community. Many authors have emphasized the need to conserve traditional medicinal knowledge to ensure the sustainable use of medicinal plants [3, 7, 13]. However, Glogov and Pavlova [14] argued that any effort at the conservation and sustainable use of medicinal plants would first require systematic documentation of such knowledge. Quantitative surveys have been identified as one of the most reliable approaches to achieve this objective as they provide the most reliable information for the conservation and value addition to resources [3, 7, 13]. Additionally, a central question in ethnobotanical studies has been what drives local people selection of medicinal plants? The versatility and ecological apparency hypotheses are usually invoked to explain plant selection for use [15]. The versatility hypothesis suggests that people are more likely to retain knowledge, use and access to a plant that has a greater number of applications for humans, while the ecological apparency hypothesis posits that local communities select nonapparent plants (i.e., short-lived or early successional plants) for medicinal use than apparent plants (i.e., perennial, woody, dominant plants) [15, 16]. The sociodemographic traits and knowledge dynamics hypothesis posit that sociodemographic traits such as age, gender, religion and literacy drive ethnobotanical knowledge dynamics. These hypotheses have rarely been tested in the global south and rare studies that tested these hypotheses found mixed results, creating a gap in our understanding of medicinal plant selection [15]. Additionally, Gaoue et al. [15] argued that the single predictor approach used in most studies in testing these hypotheses does not account for the fact that people often select plants for use based on multiple factors at the same time. Therefore, our objectives were to: (i) identify and document medicinal shrubs and herbs in the local communities around the Asukese and Amama Shelterbelt Forest Reserves; (ii) assess the relative importance of documented medicinal shrubs and herbs in the local communities around the Forest Reserves; (iii) evaluate the influence of sociodemographic traits on ethnobotanical knowledge of medicinal plants; (iv) assess the degree of uniqueness of diseases treated by medicinal plants (native vs. introduced); and (v) determine the influence of plant versatility and ecological apparency on medicinal plant selection. Transforming traditional knowledge into scientific knowledge is critical to revalue and rationally use such rich knowledge. Therefore, it is expected that this study will provide the basis for further investigation and potential discovery of novel plant-based solutions to contemporary health challenges, preserve rich indigenous knowledge on medicinal flora in the area and contribute to the achievement of good health and well-being (SDG 3). Our research framework and process is presented in Figure 1.

## 2. Methods

### 2.1. Study Area

**Figure 2:**
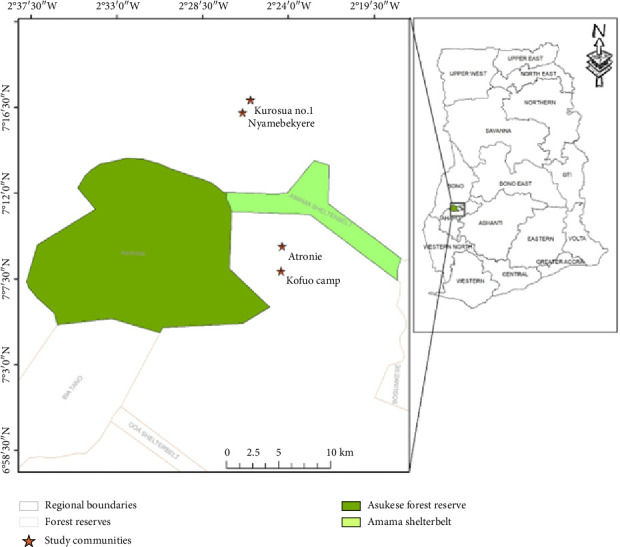
Map of the study area showing study communities.

Our study was carried out in four communities, namely, Kuffour camp, Atronie, Kurosua, and Nyamebekeyere, which are fringe communities of the Asukese and the Amama Shelterbelt Forest Reserves (Figure 2). The two forest reserves form the Forest Management Unit 17, located in the Sunyani Forest District, Bono Region, Ghana. The Asukese and the Amama Shelterbelt Forest Reserves are located between 7° 9′ 16″ N and 2° 29′ 57″ W and 7° 11′ 31″ N and 2° 21′ 57″ W, and they are classified as Moist Semi-deciduous North West Forest Type [12]. The two reserves cover an area of 309 km^2^ and are about 279–287 m above sea level. The study area has an equatorial climate and bimodal rainfall pattern: the major rainfall (1270 mm mean annual precipitation) occurring between March and September and the minor season (900 mm mean annual precipitation) occurring between October and December [13]. The mean monthly temperature ranges between 23°C and 33°C, and the mean relative humidity ranges between 75% and 80% during the rainy seasons and between 70% and 80% during the dry seasons.

The population of the study area is about 76,937, and they are primarily farmers, with cocoa, citrus, mangoes and oil palm being the major perennial cash crops and maize, yams, cassava and vegetables being the major annual cash crops. Concerning ethnicity, the residents are predominantly *Akans* and *Bonos*; a few tribes who migrated from the northern and upper regions constitute the minority. Christianity is the main religion followed by Islam and traditional religions. The local communities mainly depend on rainfall and rivers as their water source for domestic and farming activities, supplemented by pipe-borne water and wells. Fuelwood and charcoal are the primary energy sources for household activities, while the national electricity grid provides lighting. Diseases reported in the study area include malaria, anaemia, infertility, skin disorders, metabolic disorders and hypertension. The local communities rely on medicinal plants and traditional healers are their primary source of healthcare, and it is generally their first or only resort. Thus, the general welfare of the local communities in our study area and most rural communities in developing countries depend on indigenous knowledge about medicinal plants.

### 2.2. Sampling Procedure

The ethnobotanical survey was carried out between March and June 2023; March to June coincides with the major raining season which favours the availability of herbs and shrubs. A list of forest reserves in the Bono Region of Ghana was generated, and two Forest Reserves were randomly selected. Following the selection of the two forest reserves, a list of peripheral communities was generated and four communities were randomly selected. We considered residents older than 20 years and who have resided in the study area continuously for more than 10 years as being mature and knowledgeable to provide information about medicinal plants [4, 6]. Respondents in selected communities were randomly selected, while herbalist and homeopathic clinics were selected using snowball sampling [4]; in total, 78 respondents were selected. Structured and semistructured questionnaires coupled with interviews and field visits were used to conduct the ethnobotanical survey; interviews and questionnaire administration were done in Twi because it was the main local dialect in the study area and all the respondents and trained research assistants were proficient in Twi (e.g., [7, 17]). The respondents were informed about the purely academic and noncommercial nature of the study, and an informed consent was obtained before the respondents were engaged in the study. Our study complied with the Declaration of Helsinki and Tokyo for research with humans and the Ethics Committee of the University of Energy and Natural Resources. The Sunyani Forest District permitted field tours in the two Forest Reserves. The questionnaires and interviews focused on sociodemographic features, medicinal herbs and shrubs, diseases treated by cited plants, methods of preparing remedies, plant parts and condiments used, herbal remedies side effects, methods of administering herbal remedies and alternative uses of medicinal herbs and shrubs. The interview, questionnaire administration and field visit sessions lasted about 3–4 h per person.

### 2.3. Botanical Identification

Respondents provided the local names of medicinal herbs and shrubs, and these were confirmed and identified with the help of a local botanist, who was proficient in the Twi dialect. Collected voucher specimens were compared with already identified specimens to authenticate the field identification; this was done for only unknown herbs and shrubs [4].

### 2.4. Data Processing and Estimation of Quantitative Indices

We processed ethnobotanical information about medicinal herbs and shrubs using Microsoft Excel 2016. We evaluated medicinal plants used in the study area by estimating the following quantitative indices: indigenous knowledge index (IKI), relative citation frequency (RCF), relative species use report (RSUR), relative use citation frequency (RUCF), species use value (SUV), family use value (FUV), plant part value (PPV), use diversity index (UDI), informant agreement ratio (IAR) and ethnobotanical importance value (EIV).

#### 2.4.1. IKI

The IKI measures the indigenous knowledge level of an individual respondent. It was considered in this study that respondents' ability to identify medicinal plants, diseases treated, plant parts used, condiments used and side effects demonstrates their knowledge level about medicinal plants [6]. For each citation in each category, a score of one (1) was awarded and scores were summed for each respondent. Therefore, a respondent who cited more plant species, diseases treated by each mentioned plant species, plant parts per plant used to prepare remedies, condiments and side effects per plant species demonstrated greater ethnobotanical knowledge and vice versa. Thus, IKI reflects the ethnobotanical knowledge level of a respondent.

#### 2.4.2. EIV

The EIV measures how useful and predominant a plant species is for diverse medicinal and nonmedicinal purposes. We considered species with the highest EIV as the most versatile. Versatility is considered a positive selection factor for plant use in general—i.e., people are likely to retain common knowledge, use and access to a plant with diverse utility for both medicinal and nonmedicinal purposes. EIV was estimated as the sum of the RCF, RUCF and RSUR of a cited species. The RCF is the ratio of the number of respondents who mentioned a particular species to the total number of respondents expressed as a percentage (equation (1) [6, 17]). RCF reflects the common knowledge about the usefulness of a medicinal plant [4, 6], and it ranges between 0 and 100. Thus, higher RCF values mean a greater proportion of the respondents consider a particular cited species to be medically beneficial, and lower RCF values mean the opposite:

(1) $$\begin{array}{c} \text{RCF}\left(\%\right)=\frac{\text{Nr}}{N}\times 100, \end{array}$$

where Nr is the number of respondents who cited a particular species and *N* is the total number of respondents.

The RSUR is the ratio of the use reports of a particular species to the sum of the use reports of all species expressed as a percentage (equation (2)). It shows the number of different diseases a particular species is used to treat relative to the overall number of different diseases reported for all species. It reflects a plant species' value in treating diverse ailments. RSUR ranges between 0 and 100, and higher values indicate that a wider range of diseases can be treated using remedies made from the species:

(2) $$\begin{array}{c} \text{RSUR}\left(\%\right)=\frac{\text{Us}}{U}\times 100, \end{array}$$

where Us is the number of different use reports of a particular species and *U* is the sum of the use reports of all species. For example, if two respondents cited a certain species for the treatment of three different diseases and two common diseases, the total number of different use reports for the species would be five and the RSUR would be five divided by the total number of different uses reports cited by the respondents for all species multiplied by 100.

The RUCF is the ratio of the total number of uses specified for a particular plant species by all respondents to the total number of uses mentioned by all respondents for all species (equation (3)). RUCF accounts for the number of times the specified plant species has been cited for treating each of the different diseases it is used to treat. For example, if two respondents cited a certain species for the treatment of three different diseases and two common diseases, the total number of uses specified for the species would be seven and the RUCF would be seven divided by the sum of the number of uses specified for each species multiplied by 100. It indicates medicinal species which are often used for the treatment of diverse diseases:

(3) $$\begin{array}{c} \text{RUCF}\left(\%\right)=\frac{\text{UT per species}}{\text{UT}}\times 100, \end{array}$$

where UT per species is the total number of uses specified for a particular species and UT is the total number of uses mentioned by all respondents for all species.

Plant species with the highest EIV means those species are used in treating a wide range of diseases by a more significant proportion of the population. Hence, they constitute the most important plant species in terms of their medicinal value for treating several diseases compared to other medicinal plants.

#### 2.4.3. SUV and FUV

The SUV measures the value of a cited species to the local community relative to its medicinal use [4]. SUV reflects how significant a medicinal plant is to a given community [6]. It was estimated using the following equation:

(4) $$\begin{array}{c} \text{SUV}=\sum \left(\frac{\text{SUR}}{N}\right)*\text{Ns}, \end{array}$$

where SUR = species use report, which is the total number of medicinal uses provided by the respondents for a particular species, *N* = total number of respondents, and Ns = number of respondents citing a particular species for the treatment of various ailments in the community. The FUV is the ratio of the sum of the SUVs of a particular plant family to the number of species cited by the respondents which belong to that same plant family (equation (5)). FUV is an index of cultural importance which shows the medicinal relevance of plant families to a given local community [4]:

(5) $$\begin{array}{c} \text{FUV}=\frac{\Sigma \text{SUVs}}{\text{ns}}, \end{array}$$

where ∑SUVs = sum of the SUVs of all species belonging to the same plant family and ns = number of species mentioned by the respondents belonging to that same plant family.

#### 2.4.4. UDI and IAR

The UDI measures how diverse the medicinal use of a given species is in a given community. It rises with the number of diseases a species is used to treat and the proportion of respondents citing the species for treating each of the mentioned diseases. The higher the UDI, the higher the number of different ailments the species is used to treat according to a larger proportion of the respondents:

(6) $$\begin{array}{c} \text{UDI}=\frac{\text{LogSUR}}{\text{LogUCF}}, \end{array}$$

where UDI = use diversity index, SUR = species use report, and UCF = species use citation frequency. The IAR ranges between 0 and 1 and reflects the degree of consensus among respondents in the medicinal use of a specific plant species for treating particular categories of ailments. The higher the IAR, the higher the agreement in selecting species for treating specific disease categories and vice versa [6]. IAR was estimated using the following equation:

(7) $$\begin{array}{c} \text{IAR}=\frac{\text{Nc}-\text{Nd}}{\text{Nc}-1}, \end{array}$$

where Nc = citation frequency of all plant species used to treat a particular category of ailment and Nd = number of different species used in treating the cited category of ailment.

#### 2.4.5. PPV

The PPV index indicates the part of a medicinal plant that is usually harvested and used to prepare remedies for treating ailments in a given community. PPV shows which portion of the medicinal plant is the most preferred or dominant plant part for treating diseases in the communities [6]. It was calculated using the following equation:

(8) $$\begin{array}{c} \text{PPV }\left(\%\right)=\frac{\text{PPUR per part}}{\text{PPUR}}\times 100, \end{array}$$

where PPUR per plant part = sum of reported uses per a specified portion of the plant and PPUR = sum of the number of uses for all parts.

### 2.5. Data Analysis

We analysed the processed ethnobotanical information about medicinal herbs and shrubs using the Statistical Package for the Social Sciences (version 23). Linear mixed-effect regression (LMEs) was conducted to assess the influence of eight sociodemographic traits (gender, religion, age, educational level, origin status, type of family headship, marital status and number of dependents) on ethnomedicinal knowledge (IKI). Using the LMEs approach, plant versatility was modelled as a function of plant origin (native vs. introduced) and number of plant parts used with cultivation status (wild vs. cultivated) as a random variable. The final model for all LMEs analysis was selected based on Akaike's information criterion (AIC) and Schwarz's Bayesian criterion (BIC). One-way ANOVA was conducted to test whether ecological apparency (annuals vs. perennials) influenced the estimated indices; significant differences were established at *α* = 0.05. The normality of the residuals was determined by the Kolmogorov–Smirnov normality test. The Jaccard dissimilarity index was used to assess degree of uniqueness of diseases treated by medicinal plants (native vs. introduced). The Jaccard dissimilarity index was estimated as one (1) minus the ratio of diseases treated by both native and introduced species to those treated by either native plants or introduced ones as a percentage.

## 3. Results

### 3.1. Influence of Sociodemographic Characteristics on Ethnobotanical Knowledge (Sociodemographic Traits and Dynamics of Knowledge Hypothesis)

There were 1.6-fold more men involved in the use of medicinal plants than females (Appendix Table 1). Nearly 60% of the respondents were farmers. Mostly, married people (54%) were the common group, others were single (23%), and few were either widowed (14%) or divorced (9%). The participants included both the young and the aged; their ages were 24–78, with the average of 45 years. Christianity was the predominant religion (83%), and traditional religion was the least practised religion (4%). Most of the respondents had basic education (81%), while a few (5%) were educated up to the tertiary level.

Ethnomedicinal knowledge of herbs and shrubs was significantly shaped by gender, number of respondents' dependents and type of family headship (Table 1). The number of dependents a respondent had positively influenced their ethnomedicinal knowledge of herbs and shrubs. Additionally, males had higher ethnobotanical knowledge than females, while matriarchal families (MFs) had higher ethnomedicinal knowledge than patriarchal families (PFs). The primary method of ethnobotanical knowledge transfer in the local communities was through parental training (31%) followed by community information sharing (25%). Herbalists (20%) and formal training (15%) also played a significant role in ethnobotanical knowledge transfer, while the media (6%) and apprenticeship (3%) played minor roles. Respondents' age positively correlated with their ethnobotanical knowledge (*r* = 0.282; *p* = 0.012) and MFs (median age = 51.0) were relatively older than PFs (median age = 43.5).

### 3.2. Influence of Plant Versatility and Ecological Apparency on Medicinal Plant Selection

The number of plant parts used per plant was directly related to EIV (relative to both medicinal and general purposes), indicating that plant species with the most useful number of organs were the most versatile plants and hence more important ethnobotanically (Table 2). The direct relationship between the number of plant parts used per plant and EIV reveals the importance of plant versatility in meeting diverse medicinal and general needs within communities. Furthermore, contrary to our expectations, we found no significant influence of ecological apparency on medicinal plant selection, as evidenced by the similar mean RCF, RSUR, RUCF, SUV, UDI and EIV across different plant life forms (*p* > 0.05). Moreover, our analysis of the dissimilarity index revealed distinct patterns in the diseases treated by using exotic or native plants. It was found that diseases treated by using only exotic or introduced shrubs or herbs were unique from those treated by using only native ones (Jaccard dissimilarity index = 65.6%), which underscores the unique therapeutic properties and applications of each category. The number of diseases treated by using exotic plants (42.2%) was nearly twofold compared to those treated by using native species (23.3%), highlighting substantial reliance on introduced species in traditional medicinal practices.

### 3.3. Medicinal Herbs and Shrubs Species and Parts Used

**Figure 3:**
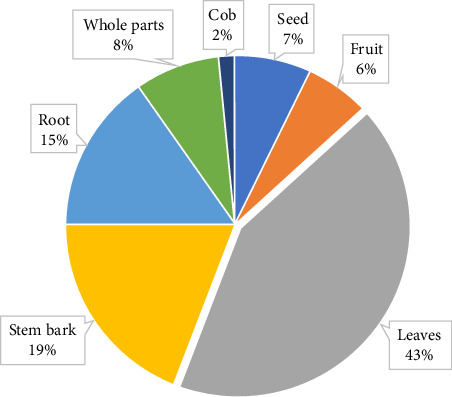
Parts of medicinal herbs and shrubs used to prepare remedies for treating various diseases by the fringe communities of the Asukese and Amama Forest Reserves.

We documented 69 medicinal herbs and shrubs used by fringe communities of Asukese and Amama Shelterbelt Forest Reserves which were efficacious in treating 101 diseases (Appendix Table 2). The 10 most frequently used plants according to their citation frequencies (CF) were *Paullinia pinnata* (CF = 32), *Momordica charantia* (CF = 19), *Chromolaena odorata* (CF = 18), *Vernonia amygdalina* (CF = 16), *Dalbergia saxatilis* (CF = 15), *Aloe cf. tenuifolia (*CF = 14), *Ocimum gratissimum* (CF = 13), *Zingiber officinale* (CF = 12), *Ananas comosus* (CF = 12) and *Alchornea cordifolia* (CF = 11). Leaves (43%) were the most preferred part, and the least was cob (2%) (Figure 3). These plant parts were sourced from one or more places which included the forest (15%), farm land (38%) and bush (47%).

### 3.4. Common Human Diseases and Health-Related Issues Treated by Documented Plant Species

**Figure 4:**
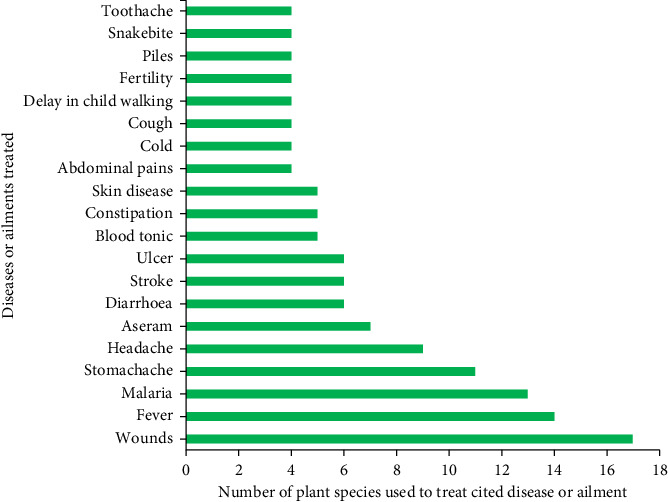
Human ailments and diseases (top 20) commonly treated by using diverse plant species.

We found that 11–17 plant species were used to treat diseases and other health issues such as wounds, fever, malaria and stomachache (Figure 4; Add. File 1). Nearly half (46%) of the diseases and other health issues were treated using two to nine different species, while half (50%) of the documented diseases were treated by using a single plant species. The medicinal shrubs and herbs were used to treat both common and specialised diseases and ailments (Figure 4). Stroke and malaria are among the top 10 causes of death in Ghana. Indigenous knowledge about frequently cited ailments and diseases indicates major healthcare issues in the study area.

### 3.5. Quantitative Evaluation of Medicinal Shrubs and Herbs

#### 3.5.1. SUV and FUV of Medicinal Shrubs and Herbs

**Figure 5:**
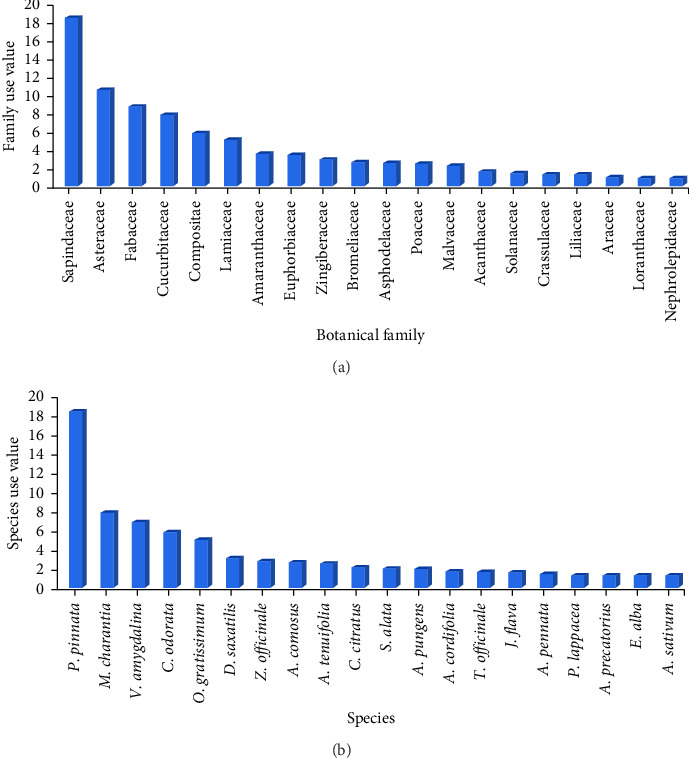
Family and species use values (top 20) of medicinal herbs and shrubs used by the fringe communities of the Asukese and Amama Forest Reserves. (a) Family use values. (b) Species use values.

Medicinal herbs and shrubs identified in the present study belonged to 35 plant families (Appendix Table 2). The most well-represented plant families in terms of the number of species used for medicinal plants followed the order: Fabaceae (9 species), Euphorbiaceae (8 species), Asteraceae (5 species), Amaranthaceae, Poaceae and Solanaceae (4 species each), Malvaceae and Zingiberaceae (3 species each) and Crassulaceae, Lamiaceae and Cucurbitaceae (2 species each). The remaining plant families had one member each. Surprisingly, some plant families such as Euphorbiaceae had a higher number of species but not higher FUV. Contrarily, there were other families with fewer species, but these plant families recorded high-use values. For example, Sapindaceae was represented by a single species, yet it recorded the highest FUV of 18.228 (Figure 5(a)). Fabaceae (8.570) recorded the second highest use value, followed by Compositae, which had only one species but had an FUV of 5.696. The other families recorded FUV less than 5.

The SUVs for the medicinal herbs and shrubs documented in this study ranged from 0.01 to 18 (Figure 5(b); top 20 species with the highest SUV shown). The species with the highest SUV was *P. pinnata* (SUV = 18.227). This was followed by *M. charantia* (SUV = 8.899), *V. amygdalina* (SUV = 6.684), *C. odorata* (SUV = 5.696), *O. gratissimum* (SUV = 4.937), *D. saxatilis* (SUV = 3.038), *Z. officinale* (SUV = 2.734), *A. comosus* (SUV = 2.582) and *A. tenuifolia* (SUV = 2.481). The remaining species had low use values (SUV < 2).

#### 3.5.2. Use Diversity and EIV of Herbs and Shrubs to Forest-Fringe Communities

The RCF ranged from 0.239 to 7.637; the top five species with the highest RCF were *P. pinnata*, *M. charantia*, *C. odorata*, *V. amygdalina* and *D. saxatilis* (Table 3). Higher RCF values indicate species the respondents share common knowledge about and consider them to be medically beneficial. The RSUR reveals medicinal species which are highly important because they can be used to treat several diseases. The species with the highest RSUR were *M. charantia*, *G. hirsutum*, *P. pinnata*, *V. amygdalina*, *A. cordifolia* and *A. pungens*. *M. charantia*, *C. odorata* and *V. amygdalina* recorded the highest RUCF. EIVs) reveal the predominant medicinal species used by most of the respondents for treating a diverse range of diseases. *M. charantia* (22.326), *P. pinnata* (20.766) and *V. amygdalina* (15.031) recorded the highest EIVs. Seven medicinal plant species, namely, *P. urinaria*, *E. hirta*, *H. floribunda*, *J. curcas*, *N. tabacum*, *B. nitida* and *R. communis*, recorded a UDI of 1.

#### 3.5.3. IAR of Respondents Relative to Medicinal Herbs and Shrubs

The IAR analyses the level of agreement among respondents regarding the use of particular medicinal plants to treat certain categories of diseases or health-related issues. The IAR of the 16 disease or health-related categories in this study ranged from 0.5 to 0.77 (Appendix Table 3). The top five categories with the highest IARs were nutritional and tonic problems (0.77), endocrine disorders (0.75), integumentary disorders (0.72), circulatory disorders (0.71) and respiratory disorders (0.70). Dental problems and developmental delays recorded the least IARs (0.5).

### 3.6. Mode of Preparation and Administration of Plant Medicine

**Figure 6:**
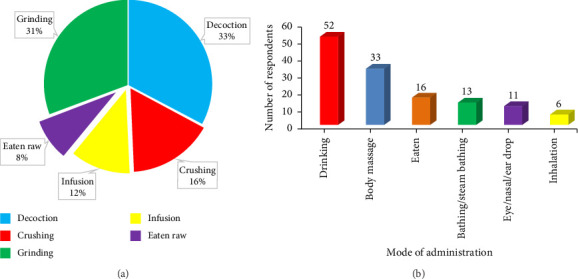
Method of preparing (panel (a) and routes of administering) (panel (b) medicinal herbs and shrubs used by the fringe communities of the Asukese and Amama Forest Reserves). (a) Mode of recipe preparation. (b) Mode of administering prepared medicinal recipes.

The methods used in the preparation of herbal remedies included grinding, crushing, infusion, decoction and eaten raw (Figure 6(a)), while the methods used to administer the prepared remedies to patients included drinking, body massage, bathing or steam bathing, eating, inhalation and eye, ear or nasal drop (Figure 6(b)). Decoction (33%) and the oral route (68%) were the predominant ways to prepare and administer herbal remedies in the study area. The diverse methods of remedy preparation and administration depend on several factors including the location of the disease, severity, part of the plant used and ease of extraction.

## 4. Discussion

### 4.1. Influence of Sociodemographic Characteristics on Ethnobotanical Knowledge

The study revealed that ethnobotanical knowledge dynamics were shaped by gender, type of family headship and number of dependents of a respondent (Table 1). We found that even though men had greater ethnobotanical knowledge than women, MFs had greater ethnobotanical knowledge. This observation is possibly because ethnobotanical knowledge positively correlated with age (*r* = 0.282; *p* = 0.012) and MFs were relatively older (MF median age 51 vs. PF median age 43). Furthermore, women are reportedly familiar with garden herbs and herbaceous forest plants used as medicines [14] because they take care of the home. This knowledge may be passed on to other family members through a practical application or home training which was reported as the highest means of plant knowledge transfer in the survey. That notwithstanding, other studies attribute greater ethnobotanical knowledge among MFs to homogeneity of knowledge in women, their ability to be familiarized with more useful species and their demonstration of better knowledge sharing than men [14, 18], but these were not explored in the current study. In-depth knowledge about the surrounding flora is crucial to using them for food, medicine and other domestic or commercial uses [19]. Therefore, the positive influence of number of a respondent's dependents on ethnobotanical knowledge may be due to economic dependency on natural resources which may incentivize individuals to enhance their ethnobotanical knowledge in order to optimize resource utilization and meet family needs [19]. Ethnobotanical knowledge was however independent of sociodemographic characteristics such as educational level, origin of respondent and marital status [20]. Similar to other findings [e.g., 24], ethnobotanical knowledge was not influenced by a respondent's origin possibly because immigrant migrated from areas with similar vegetations and some may have stayed in the study communities for longer periods and are therefore conversant with the plants around them [21]. In general, we accept the hypothesis that ethnobotanical knowledge dynamics were shaped by sociodemographic traits.

We found that there were 1.6-fold more men involved in the use of medicinal plants than females which is consistent with previous studies [e.g., 13; 7]. Male dominance in the use of medicinal plants could be explained by women's engagement in domestic activities that keeps them busy and the location of most medicinal plants in the forest and wild which make it easier for men to access than women [13, 19]. Similar to Mesfin et al. [22] and Kidane et al. [23], parental training was the most common mode of ethnobotanical knowledge transmission in the study communities.

### 4.2. Influence of Plant Versatility and Ecological Apparency on Medicinal Plant Selection

The study revealed that plants with the most number of useful organs were the most versatile (Table 2). We propose two hypotheses based on our data and existing literature to explain this finding. Firstly, plants with higher number of useful parts may have greater cultural significance (personal field observation) and traditional knowledge associated with their use (*r* = 0.439; *p* < 0.001). This is consistent with existing literature which suggests the selection of medicinal plants is influenced by cultural and traditional knowledge [15]. Therefore, plants with multiple useful organs may have been discovered and valued over generations due to their versatility in addressing a range of medicinal and nonmedicinal needs. Thus, the finding may reflect the central role of versatile plant species in traditional practices. Secondly, plants with a higher number of useful parts may contain a greater diversity of bioactive compounds, leading to a broader spectrum of pharmacological effects. For example, our data and existing literature suggests that remedies are often prepared from different plant parts and condiments (mostly other plant parts or products) are often added to produce synergistic effects or used separately to meet different health needs (Appendix Table 2; 27; 3; 6). Thus, medicinal plant selection may prioritize species with diverse chemical profiles to address a range of health issues. Therefore, our finding of a positive influence of number of useful parts on versatility may be linked to potential chemical diversity and synergistic opportunities, but urgent research is need to establish this. The fact that plants with the most number of useful organs were the most versatile is promising as it may enhance conservation and offer more sustainable economic opportunities. For example, harvesting multiple plant parts from a single species can contribute to conservation and economic sustainability since it allows for the utilization of different plant components without depleting the entire population [15].

Contrary to the findings of Acosta et al. [24], we found no support for the ecological apparency hypothesis. This is possibly because the selection of medicinal plants is influenced by several factors including sociodemographic, biophysical and phytochemical properties (Table 1; [7, 13, 15, 25]). This calls for a holistic approach to biodiversity conservation that considers both ecological and cultural dimensions. Our data, however, show that diseases treated by using only native plants were uniquely different from those treated by using only introduced species which possibly supports the diversification hypothesis, even though it was not directly tested. The fact that 38 diseases were treated by using only introduced or exotic plants while 21 diseases were treated by using only native plants possibly suggests that introduced or exotic and native medicinal plants were selected to improve the diversity of plants used to treat the range of diseases in the communities [26]. That notwithstanding, further studies on medicinal plant selection and their phytochemistry are urgently needed.

### 4.3. Medicinal Herbs and Shrubs Species and Parts Used

The most cited species was *P. pinnata*. Studies on *P. pinnata* have revealed that the plant possesses high antioxidant properties due to the presence of phytochemical groups such as saponins, tannins, terpenoids, alkaloids, cardiac glycosides, flavonoids, anthraquinones, polyphenols and triterpenes [27, 28]. Pharmacological properties like antihypertensive, anticancer, anticholesterol, cardiotonic, analgesic and anti-inflammatory have also been associated with this plant [27, 29]. Furthermore, *P. pinnata* has been found to possess antimalarial, antidiarrhoea, anticonvulsive [28] and antidepressant-like effects [30]. These reasons could justify the relatively high use of the plant. Other highly cited plants were *M. charantia, C. odorata, V. amygdalina, D. saxatilis, A. tenuifolia, O. gratissimum, Z. officinale*, *A. comosus* and *A. cordifolia*. Most of these species have also been reported in Ghana [3] and other parts of the world to be most preferable and effective in curing diseases. The greater citation frequency of these medicinal plants may indicate the need to conserve these herbs and shrubs species to prevent their extinction since a more significant population harvests them for use. Also, in-depth pharmacological and phytochemical assessment of the species are needed.

Consistent with several studies, leaves (43%) were the most predominant plant part use to prepare remedies [1, 3, 6, 31]. Photosynthetic activities and richness of secondary metabolites in the leaves and the minimum stress involved in collecting them may explain their frequent use in disease treatment [17]. Most importantly, harvesting of the leaves of plants is more sustainable as it causes less damage to plants than harvesting other parts like the roots and stem which may result in plant death [31, 32]. Similar to previous authors (e.g., [33]), the medicinal herbs and shrubs parts were predominantly sourced from the forest. The richness of diverse forms of plant species inhabiting the forest possibly accounted for the surge in the collection of herbs and shrubs from this location [34]. As such, it is important to give attention to issues related to the sustainability and conservation of forest plant species because overexploitation may threaten their future existence [19]. The use of plant leaves can be promoted as a suitable alternative when all plant parts have similar pharmacological activities [32, 33].

### 4.4. Common Human Diseases and Health-Related Issues Treated by Documented Plant Species

Wounds, fever, malaria and stomachache were the top four most prevalent diseases or health issues in the study area, and their prevalence may reflect the respondents' occupational risks or social context. For example, cuts and stabs are reported to be common among rural farming communities possibly due to the lack of appropriate farming gear [35]. The study revealed that 11–17 medicinal herbs and shrubs were used to treat or address the most prevalent human diseases or health issues in the study area. Similar findings have been reported in other parts of Ghana [1, 3, 13] where multiple plants were cited for these diseases. The high number of species assigned to treating these diseases can be attributed to their prevalence in the study area, thereby causing people to pay attention to discovering remedies to curb them. Malaria, for instance, is a life-threatening disease and one of the leading causes of death across the globe, with an estimated case of about 247 million [36]. Furthermore, it is a major cause of mortality in Ghana, especially among children under 5 years [36]. Many authors argue that traditional medicine has gained roots in malaria treatment, especially in rural areas globally and in Ghana due to their affordability, accessibility and the high cost that accompanies modern treatment [3, 7]. Notably, nearly half (46%) of the diseases and health-related issues were treated using two to nine different species, while half (50%) of the documented diseases were treated by using a single plant species. This finding is consistent with other studies [17]. Knowledge of various ailments indicates significant health problems in the area, which are greatly important to the health service.

### 4.5. Quantitative Evaluation of Medicinal Shrubs and Herbs

#### 4.5.1. SUV and FUV of Medicinal Shrubs and Herbs

We found 67 species, belonging to 35 families used for treating various diseases in the study area. The most well-represented plant families in terms of the number of species were Fabaceae, Euphorbiaceae and Asteraceae, which corroborates studies in Ghana and elsewhere [3, 7, 21, 32, 37]. According to Chaachouay et al. [32], the dominance of medicinal plants in families such as Fabaceae, Euphorbiaceae and Asteraceae could be due to the abundance of secondary metabolites present in the plant's species belonging to the families, their wide distribution or easy accessibility [32]. For example, plant species from Fabaceae family are known to be rich in phytochemicals such as phenols and tannins and they can tolerate a wide range of environmental conditions which may account for their wide distribution across Africa [33].

Use values of medicinal plants usually imply their relative importance to the people [3]. The highest FUVs were recorded for the families Sapindaceae, Fabaceae and Asteraceae (Figure 5(a)), suggesting these plant families were the most relevant to the local communities. The SUVs for the documented herbs and shrubs in the present study ranged from 0.01 to 18 (Figure 6(b)). We found that *P. pinnata* was the most important medicinal plant in the study area with the highest SUV (Figure 5(b)). Other top species, according to the SUV, were *M. charantia* and *V. amygdalina*. Plant species with higher use values are usually used to treat a broad spectrum of diseases [38]. For instance, leaf decoction of *P. pinnata* was used by the study communities to treat a variety of diseases such as stroke, stomach ulcer, miscarriage, erectile disfunction, snake bite and waist pain. Species with high-use values need conservation measures to ensure their sustainability [38].

#### 4.5.2. Use Diversity and Importance Value of Herbs and Shrubs in Forest-Fringe Communities

We found that 33% of the documented species had a UDI greater than 0.6 (Table 3), suggesting that a more significant proportion of the medicinal plants were used to treat different diseases and address health issues, with high consensus among the respondents. This may be attributable to the fact that they are efficacious in the treatment of several kinds of diseases due to the active ingredients such as alkaloids, tannins, flavonoids, saponins, phenols, cardiac glycosides, terpenoids and steroids present in them [27, 33, 39], but urgent studies on the phytochemistry and efficacy of these plants are needed. The RCF values ranged from 0.239 to 7.637 with *P. pinnata*, *M. charantia* and *C. odorata* recording the highest values. The high RCF recorded for these plants indicates their high use in the area [40]. Higher RCF values of species also indicates their large acceptance by the respondents who share common knowledge about these species and consider them medically beneficial [32, 40]. Also, the species with the highest RSUR were *M. charantia*, *G. hirsutum* and *P. pinnata*, suggesting these medicinal species are highly important because they can be used to treat several diseases. Again, *M. charantia*, *C. odorata* and *V. amygdalina* recorded the highest RUCF. EIVs, which reveal the predominant medicinal species used by most respondents for treating a diverse range of diseases, were highest for *M. charantia*, *P. pinnata* and *V. amygdalina*. The consistency of use and importance given to these plants suggests their prominent place in the healthcare of the people and highlights the importance of traditional knowledge [40]. Moreover, the inexpensive accessibility and availability of these species, as well as their pharmacological properties and bioactive constituents, is congruent to their preference. As such, it is imperative to include them in appropriate conservation strategies to prevent any future threat [41].

#### 4.5.3. IAR of Respondents Relative to Medicinal Herbs and Shrubs

The diseases or health issues mentioned by the respondents were first classified into 16 categories from which the IAR was calculated. The IAR of the 16 disease or health-related categories in this study ranged from 0.5 to 0.77 (Appendix Table 3). However, this range is lower than those reported by Ssenku et al. [37] in Uganda and Tantengco [38] in the Philippines. The difference in IAR values could be as a result of the differences in disease prevalence and the diversity and availability of medicinal herbs and shrubs. With the exception of dental problems and developmental delays which recorded low IAR (0.5), the remaining disease or health issue categories had high IAR. The high IAR values reveal that there is high agreement regarding the use and effectiveness of herbs and shrubs in treating various disease categories in the study communities [37]. The highest IAR value (0.77) in the present study was recorded for nutritional and tonic problems. The respondents cited seven plants used for treating this disease category, but the most commonly used plant was *T. officinale*. Studies have attested to the nutritional importance of the plant due to the high levels of vitamins, minerals, proteins, essential fatty acids and fibre it contains [42]. Furthermore, antioxidant, anti-inflammatory, antimicrobial and antidiabetic properties have been associated with the plant. *T. officinale* is also rich in phytochemicals such as tannins, terpenes, flavonoids, steroids and phenolic acids [42, 43]. These essential plant constituents could have contributed to its preferred use as a vegetable in most homes and also in solving nutritional and tonic problems in the study area [43]. Essentially, species with high IAR could be sign posts to the discovery of novel drugs and therefore should be prioritized for further pharmacological and phytochemical assessment. On the other hand, the low IAR observed in dental problems and developmental delays could be attributed to the lack of equipment and expertise in diagnosing these diseases and the misconceptions surrounding developmental delays in most parts of Africa [37, 44]. This could also be linked to the differences in the sources of ethnobotanical knowledge as well as the unwillingness of some respondents to disclose which lead to the variations in species cited for certain disease categories [37].

### 4.6. Implications for Biodiversity Conservation

Biodiversity conservation is imperative because it is the source of primary health to 80% of the world's population [2, 17, 39]. It potentially increases the range of treatment options available in the forest-fringe communities [20, 45]. For example, introduced species were used to meet unique therapeutic needs, which implies conservation efforts must include introduced species, not just native ones. Therefore, we argue that linking biodiversity conservation and health may potentially stimulate people's interest in biodiversity, even though we did not directly explore this in the present study. In relation to the parts of plants used to prepare remedies, roots or stem bark constituted 34% (Figure 3); the exploitation of these plant parts reduces their potential to survive [1, 32]. This implies management interventions that discourage the use of these plant parts are essential to enhancing biodiversity conservation in forest-fringe communities. The use of diverse medicinal plants to treat predominant diseases and ailments in the study area may lead to reduced pressure on overexploited ones [19, 45].

## 5. Conclusion

The ethnobotanical knowledge of herbs and shrubs among forest-fringe communities was linked to sociodemographic traits, with gender, family structure and household dynamics as pivotal determinants in the transmission and utilization of medicinal flora. Furthermore, our study demonstrates that plants with multiple useful parts exhibit remarkable versality in traditional medicinal practices, providing support for the plant versality hypothesis. However, we found no support for the ecological apparency hypothesis in relation to plant selection in ethnobotanical contexts. Rich and diverse medicinal flora, comprising 2–17 species each, played a pivotal role in treating a wide array of diseases and ailments prevalent in rural settings. The prominence of health issues such as wounds, fever, malaria, stomachache and headache is indicative of the health issues, social contexts and occupational risks of rural folks, highlighting the need for tailored social and healthcare interventions. Our findings shed light on gender disparities in the utilization of medicinal plants, with men (60% higher) primarily driving their use. However, MF structures emerged as key repositories of ethnobotanical knowledge, suggesting the potential for women to play a central role in its preservation and transmission. Furthermore, community-based information sharing and intergenerational transfer mechanisms are crucial for ensuring the continuity of this invaluable knowledge. Forest-fringe communities relied on exotic plants for the treatment of diseases (42.2%) than native species (23.3%), highlighting the role of exotic plants in traditional medicinal practices. Generally, exotic plants provided medicinal treatment for diseases distinct from those treated by native ones (Jaccard dissimilarity index = 65.6%), suggesting that the selection of medicinal plants may be linked to phytochemistry. Notably, families such as Fabaceae, Euphorbiaceae and Sapindaceae emerged as significant sources of medicinal flora, with species like *P. pinnata* recording the highest SUV (SUV = 18.227) and *M. charantia* (EIV = 22.326) being the predominant medicinal species used by most respondents for the treatment of a diverse range of diseases. Therefore, phytochemical and pharmacological assessment of plants for relevant bioactive compounds can target these plant families and species. We concluded that efforts to safeguard traditional knowledge and conserve biodiversity must prioritize the holistic integration of both native and exotic medicinal plants, thereby ensuring the continued resilience and health of forest-fringe communities [46–48].

## Figures and Tables

**Table 1:** Influence of sociodemographic characteristics on ethnobotanical knowledge in the fringe communities of Asukese and Amama Forest Reserves.

Parameter	Mean (SEM)^n^	Slope (SEM)/intercept	df	*p* value
Intercept		7.80 ± 3.11^i^	53.63	0.015
Dependents' number	4.13 (na)	0.68 ± 0.16^s^	65.00	< 0.001
HeadshipType = matriarchal	18.72 ± 2.14^a^	6.36 ± 2.03^i^	65.36	0.003
HeadshipType = patriarchal	12.35 ± 1.15^b^			
Gender = Male	17.29 ± 1.90^a^	3.51 ± 1.63^i^	65.94	0.035
Gender = Female	13.78 ± 1.59^b^			
Family size	5.75 (na)	−0.21 ± 0.18^s^	65.65	0.231
Religion = Traditionalist	17.40 ± 3.77^a^	3.39 ± 4.27^i^	65.89	0.431
Religion = Christian	15.20 ± 1.00^a^	1.19 ± 2.02^i^	65.69	0.559
Religion = Muslim	14.01 ± 2.01^a^			
UserGroup = Trader or seller	16.19 ± 2.07^a^	0.35 ± 2.18^i^	65.24	0.873
UserGroup = Farmer	14.58 ± 1.64^a^	−1.25 ± 1.66^i^	65.07	0.453
UserGroup = Herbalist	15.84 ± 1.94^a^			

*Note*: *i* = intercept estimate and s = slope estimate. ‘n' = mean values with the same letters are similar and those with different values are significantly different. na = mean standard error estimates not applicable. Parameter levels with different letters indicate significant mean difference while those with similar letters indicate no significant mean difference.

Abbreviation: SEM, standard error mean.

**Table 2:** Influence of versatility on ethnobotanical importance value (EIV).

Dependent variable	Parameter	Mean (SEM)^n^	Slope (SEM)/intercept	df	*p* value
Ethnobotanical importance value (medicinal)	Intercept		3.20 ± 1.32^i^	66	0.018
	Plant parts used number	1.71 (na)	1.84 ± 0.64^s^	66	0.005
	Originality = Native	5.22 ± 0.75^a^	−1.13 ± 0.99^i^	66	0.257
	Originality = Introduced	6.34 ± 0.63^a^			

Ethnobotanical importance value (general use)	Intercept		3.63 ± 1.35^i^	66	0.009
	Plant parts used number	1.71 (na)	2.08 ± 0.65^s^	66	0.002
	Originality = Native	6.05 ± 0.76^a^	−1.14 ± 1.00^i^	66	0.261
	Originality = Introduced	7.19 ± 0.65^a^			

*Note: i* = intercept estimate and s = slope estimate. ‘n' = mean values with the same letters are similar and those with different values are significantly different. na = mean standard error estimates not applicable. Parameter levels with different letters indicate significant mean difference while those with similar letters indicate no significant mean difference.

Abbreviation: SEM, standard error mean.

**Table 3:** Quantitative evaluation of medicinal herbs and shrubs used by the fringe communities of the Asukese and Amama Forest Reserves based on selected indices.

Vernacular name	Species	Source of collection	PPUN	RNMUR	RCF	RSUR	RUCF	SUV	EIV	EIVg	UDI
Nkruma/Okro	*Abelmoschus esculentus* Moench.	Farms	2	5	0.48	1.98	0.48	0.08	2.94	3.94	0.63
Nyame eni	*Abrus precatorius* L.	Forest, bush, farms	2	15	2.15	1.98	1.76	1.25	5.89	8.89	0.29
Nwere	*Acacia pennata* Willd.	Forest, bush, farms	2	10	2.15	1.98	1.92	1.37	6.05	8.05	0.28
Efomwisa	*Aframomum melegueta* K.Schum.	Bush, farms	1	5	0.48	0.99	0.32	0.05	1.79	2.79	0.00
Guakro	*Ageratum conyzoides* L.	Bush	3	0	0.95	2.97	0.96	0.30	4.88	4.88	0.61
Gyama/Ogyama	*Alchornea cordifolia* (Schumach. & Thonn.) Müll.Arg.	Bush, forest, farms	3	10	2.63	5.94	1.92	1.67	10.48	12.48	0.72
Gyeene Kankan	*Allium sativum* L.	Farms	2	10	1.91	3.96	1.92	1.22	7.79	9.79	0.56
Aloe vera	*Aloe cf. tenuifolia* Lam.	Bush, farms	1	5	3.34	2.97	2.24	2.48	8.55	9.55	0.42
Nsoesoe/Nkaseenkasee	*Alternanthera pungens* Kunth	Bush, farms	3	0	2.15	5.94	2.72	1.94	10.80	10.80	0.63
Nantwibini	*Amaranthus spinosus* L.	Bush, forest	2	0	0.72	3.96	0.96	0.23	5.63	5.63	0.77
Aborobe	*Ananas comosus* (L.) Merr.	Bush, farms	3	10	2.86	3.96	2.72	2.58	9.54	11.54	0.49
Asparagus dua	*Asparagus africanus* L.	Forest	1	5	0.24	0.99	0.16	0.01	1.39	2.39	0.00
Odwene/Aduma	*Baphia nitida* Lodd.	Forest	2	5	0.24	1.98	0.32	0.03	2.54	3.54	1.00
Dwirentwi/Gyinantwi	*Bidens pilosa* L.	Forest, bush	2	0	1.19	4.95	1.12	0.44	7.26	7.26	0.83
Nkokodwe	*Boerhavia diffusa* L.	Farms	1	0	0.24	0.99	0.16	0.01	1.39	1.39	0.00
Taa meawu	*Brachyachne obtusiflora* (Benth.) C.E. Hubb.	Bush	1	5	0.48	1.98	0.48	0.08	2.94	3.94	0.63
Kwaebese	*Bryophyllum pinnatum* (Lam.) Oken	Bush, farms	2	0	1.67	2.97	1.44	0.80	6.08	6.08	0.50
Brofere	*Carica papaya* L.	Farms	1	5	0.24	0.99	0.16	0.01	1.39	2.39	0.00
Akyeampong	*Chromolaena odorata* (L.) R.M.King & H.Rob.	Bush, forest	1	0	4.30	3.96	3.99	5.70	12.25	12.25	0.43
Hwiremoo	*Combretum smeathmannii* G. Don.	Bush, forest, farms	1	5	0.95	1.98	0.96	0.30	3.89	4.89	0.39
Ti-ahaban	*Cymbopogon citratus* (DC.) Stapf	Forest	1	5	2.39	3.96	2.56	2.03	8.90	9.90	0.50
Ahomakyem	*Dalbergia saxatilis* Hook.f.	Bush, forest, farms	4	5	3.58	2.97	2.56	3.04	9.11	10.11	0.40
Ntum	*Eclipta alba* Hassk.	Bush, forest, farms	1	0	1.67	3.96	2.24	1.24	7.87	7.87	0.53
Kakaweadwe	*Euphorbia hirta* L.	Bush, farms	1	0	0.48	1.98	0.32	0.05	2.78	2.78	1.00
Asaawa	*Gossypium hirsutum* L.	Bush, farms	3	10	1.43	6.93	1.92	0.91	10.28	12.28	0.78
Kagya	*Griffonia simplicifolia* (DC.) Baill.	Bush	1	0	0.72	1.98	0.64	0.15	3.34	3.34	0.50
Akokotuatu/Akomfemtikoro/Akomfem Atiko	*Heliotropium indicum* L.	Bush, farms	1	0	1.43	1.98	0.96	0.46	4.37	4.37	0.39
Sesame	*Holarrhena floribunda* (G.Don.) Dur.& Schinz	Forest, bush	2	5	0.48	1.98	0.32	0.05	2.78	3.78	1.00
Nunum nini	*Hoslundia opposita* Vahl.	Bush, farms	2	0	0.48	0.99	0.32	0.05	1.79	1.79	0.00
Nkradedua	*Jatropha curcas* L.	Bush, farms	3	5	0.48	1.98	0.32	0.05	2.78	3.78	1.00
Afama	*Justicia flava* Vahl.	Bush, forest, farms	1	5	1.91	3.96	2.40	1.52	8.27	9.27	0.51
Egorɔ	*Kalanchoe integra* Kuntze.	Forest, bush	2	10	0.95	3.96	1.44	0.46	6.35	8.35	0.63
Ananse dokono	*Lantana camara* L.	Forest	1	0	0.24	0.99	0.16	0.01	1.39	1.39	0.00
Bankye	*Manihot esculenta* Crantz	Farms	2	10	1.43	4.95	1.28	0.61	7.66	9.66	0.77
Odubrafo	*Mareya micrantha* (Benth.) Müll.Arg.	Bush, forest, farms	1	5	1.67	2.97	1.12	0.62	5.76	6.76	0.56
Mfofo	*Melanthera scandens* Schu, Nach & Thonn	Bush	2	0	0.95	0.99	0.64	0.20	2.58	2.58	0.00
Militia	*Millettia ferruginea* (Hochst) Baker	Bush	1	5	0.24	0.99	0.16	0.01	1.39	2.39	0.00
Mumuanka	*Mimosa pudica* L.	Bush	1	0	0.24	0.99	0.16	0.01	1.39	1.39	0.00
Nyanya	*Momordica charantia* L.	Bush, farms	3	5	4.53	11.88	5.91	8.90	22.33	23.33	0.69
Dunsinkro	*Momordica foetida* Schumach	Bush	1	0	0.24	0.99	0.16	0.01	1.39	1.39	0.00
Brodeε	*Musa paradisiaca* L.	Bush, farms	2	5	1.19	2.97	1.12	0.44	5.28	6.28	0.56
Damerama	*Mussaenda erythrophylla* Schumach. and Thonn.	Bush, farms	1	5	1.67	1.98	1.12	0.62	4.77	5.77	0.36
Aya	*Nephrolepis biserrata* (Sw.) Schott	Bush, forest, farms	2	5	1.67	1.98	1.44	0.80	5.09	6.09	0.32
Bonto	*Nicotiana tabacum* Linnaeus	Forest, bush	2	10	0.48	1.98	0.32	0.05	2.78	4.78	1.00
Nunum	*Ocimum gratissimum* L.	Bush, farms	1	0	3.10	4.95	4.79	4.94	12.85	12.85	0.47
Duawusa	*Pachypodanthium* staudtii Engl. & Diels	Forest	2	0	0.24	0.99	0.16	0.01	1.39	1.39	0.00
Abakamo	*Parquetina nigrescens* (Afzel). Bullock	Forest, bush	2	5	0.72	3.96	0.80	0.19	5.48	6.48	0.86
Toa ntini	*Paullinia pinnata* L.	Bush, forest, farms	1	0	7.64	5.94	7.19	18.23	20.77	20.77	0.47
Awobe	*Phyllanthus muellerianus* (Kuntze.) Exell.	Forest, bush	1	10	0.72	0.99	0.48	0.11	2.19	4.19	0.00
Bowomaguwakyi/Awommaguwakyi	*Phyllanthus urinaria* L.	Bush, farms	2	0	0.72	2.97	0.48	0.11	4.17	4.17	1.00
Mayaabea	*Pteridium esculentum* Lucidcentral	Bush	1	0	0.48	0.99	0.32	0.05	1.79	1.79	0.00
Aposompo	*Pupalia lappacea* (L.) A Juss	Bush, farms	1	0	2.15	2.97	1.76	1.25	6.88	6.88	0.46
Adedenkruma	*Ricinus communis* L.	Bush	1	0	0.24	1.98	0.32	0.03	2.54	2.54	1.00
Ahwedeε	*Saccharum officinarum* L.	Bush, farms	1	10	0.95	0.99	0.64	0.20	2.58	4.58	0.00
Sempe	*Senna alata* (L.) Roxb.	Forest, bush	1	10	1.67	3.96	3.51	1.95	9.15	11.15	0.45
Nkwadaa brodee	*Senna occidentalis* (L.) Link	Forest, bush, farms	3	5	1.43	4.95	1.60	0.76	7.98	8.98	0.70
Sesame	*Sesamum indicum* L.	Forest, bush	1	5	0.95	1.98	0.64	0.20	3.57	4.57	0.50
Tweta	*Sida acuta* Burm. f.	Bush	2	0	1.67	2.97	2.08	1.15	6.72	6.72	0.43
Pepediewuo	*Solanum erianthum* D. Don	Forest, bush, farms	1	5	1.43	1.98	1.60	0.76	5.01	6.01	0.30
Ntoose, Nsusuwa, Asamantrowa	*Solanum lycopersicum* L.	Farms	2	10	0.95	3.96	0.80	0.25	5.71	7.71	0.86
Osisiriw/Akuakua nisuo	*Solanum torvum* Sw.	Bush, farms	2	5	0.95	2.97	0.96	0.30	4.88	5.88	0.61
Aduro kokoo/Kramankote	*Sphenocentrum jollyanum* Pierre	Bush	2	5	1.43	1.98	0.96	0.46	4.37	5.37	0.39
Wawa bima/Akotoben	*Tapinanthus bangwensis* (Engl.& K. Krause) Danser	Forest, bush, farms	2	0	1.43	4.95	1.76	0.84	8.14	8.14	0.67
Nkrangyedua	*Taraxacum officinale* F.H.Wigg.	Farms	2	5	2.15	2.97	2.24	1.59	7.35	8.35	0.42
Awonwone	*Vernonia amygdalina* Delile	Bush, farms	3	5	3.82	5.94	5.27	6.68	15.03	16.03	0.51
Mankani	*Xanthosoma mafaffa* Schott	Farms	1	5	1.67	3.96	1.76	0.97	7.39	8.39	0.58
Aburoo	*Zea mays* L.	Farms	2	0	0.48	1.98	0.64	0.10	3.10	3.10	0.50
Akakaduro	*Zingiber officinale* Roscoe	Farms	3	10	2.86	4.95	2.88	2.73	10.69	12.69	0.56
Awapuhi	*Zingiber zerumbet* L. Sm	Forest	1	5	0.24	0.99	0.16	0.01	1.39	2.39	0.00

*Note:* PPUN = number of plant parts used and EIVg = ethnobotanical importance value, including general uses.

Abbreviations: EIV, ethnobotanical importance value; RCF, relative citation frequency; RNMUR, relative nonmedicinal use report; RSUR, relative species use report; RUCF, relative use citation frequency; SUV, species use value; UDI, use diversity index.

## Data Availability

The datasets supporting the conclusions of this article are included within the article (and its additional file(s)). Further information about the data will be made available upon request.

## Supporting Information

Supporting Information 1 Appendix Table 1: Sociodemographic characteristics of respondents of the fringe communities of Asukese and Amama Forest Reserves.

Supporting Information 2 Appendix Table 2: Results on ethnobotanical uses of cited shrubs and herbs among forest-fringe communities of Ghana. Data contain the vernacular names, botanical names and methods of preparing and administering remedies.

Supporting Information 3 Appendix Table 3: Informant agreement ratio values by categories for treating various diseases in the fringe communities of Asukese and Amama Shelterbelt Forest Reserves.
