# Supplementary material for: Ethnobotanical Study of Medicinal Shrubs and Herbs Used by Forest-Fringe Communities of Ghana
Source: Scientifica (Cairo). 2025 May 19;2025:1362301. doi: 10.1155/sci5/1362301 (PMC12105890; doi:10.1155/sci5/1362301)
Supplement: Supporting Information 1 — Appendix Table 1: Sociodemographic characteristics of respondents of the fringe communities of Asukese and Amama Forest Reserves. [file 1362301.f1.docx]

**Appendix Table 1. Socio-demographic characteristics of respondents of the fringe communities of Asukese and Amama Forest Reserves**

| **Variable** | **Category** | **Total** | **Percentage (%)** |
| --- | --- | --- | --- |
| Gender | Male | 48 | 62 |
|  | Female | 30 | 38 |
| Marital Status | Married | 42 | 54 |
|  | Single | 18 | 23 |
|  | Widowed | 11 | 14 |
|  | Divorced | 7 | 9 |
| Educational Level | Primary | 16 | 21 |
|  | JHS | 24 | 31 |
|  | SHS/TEC/VOC | 23 | 29 |
|  | Tertiary | 4 | 5 |
|  | No formal education | 11 | 14 |
| Religion | Christian | 65 | 83 |
|  | Muslim | 10 | 13 |
|  | Traditionalist | 3 | 4 |
| Respondent Category | Farmer | 48 | 59 |
|  | Trader/seller | 14 | 18 |
|  | Herbalist | 18 | 23 |
| Native or Immigrant | Native | 54 | 69 |
|  | Immigrant | 24 | 31 |
| Type of family headship | Female-headed | 15 | 19 |
|  | Male-headed | 62 | 81 |

NB: *JHS = Junior high school, SHS/TEC/VOC = Senior High School/Technical Education Certificate/Vocational training.*
