# Supplementary material for: Ethnobotanical Study of Medicinal Shrubs and Herbs Used by Forest-Fringe Communities of Ghana
Source: Scientifica (Cairo). 2025 May 19;2025:1362301. doi: 10.1155/sci5/1362301 (PMC12105890; doi:10.1155/sci5/1362301)
Supplement: Supporting Information 2 — Appendix Table 2: Results on ethnobotanical uses of cited shrubs and herbs among forest-fringe communities of Ghana. Data contain the vernacular names, botanical names and methods of preparing and administering remedies. [file 1362301.f2.docx]

**Appendix Table 2. Medicinal herbs and shrubs used by fringe communities of** **Asukese and Amama Forest Reserves of Southwestern Ghana**

| **Vernacular name** | **Species** | **Family** | **PPU** | **Disease** | **MOP** | **MOA** | **Condiments** | **Side effect** |
| --- | --- | --- | --- | --- | --- | --- | --- | --- |
| Nkruma/ Okro | *Abelmoschus esculentus*Moench. | Malvaceae | F, S | Wound, Child delivery | Grinding, Infusion | Body massage | Shea butter, Ashes | None |
| Nyame eni | *Abrus precatorius L.* | Fabaceae | S, L | Epilepsy, Fever | Grinding, Decoction | Drinking, Eaten | Honey, Ginger, Powdered charcoal, Food | Weakness, Frequent running, Allergic reaction, |
| Nwere | *Acacia pennata*Willd. | Fabaceae | SB, L | Wound, Skin disease | Grinding, Decoction | Drinking, Body massage | Mahogany, Ginger | None |
| Efom wisa | *Aframomum melegueta*K.Schum | Zingiberaceae | S | Convulsion | Crushing, Grinding | Drinking, Body massage | None | None |
| Guakro | *Ageratum conyzoides*L. | Asteraceae | L, R, WP | Eye disease, Constipation, Pregnancy care | Grinding, Crushing, Decoction | Ear or eye drop, Drinking | Clay | Over bleeding |
| Gyama/ Ogyama | *Alchornea cordifolia* (Schumach. and Thonn.) Müll.Arg. | Euphorbiaceae | R, SB, L | Jaundice, Abortion, Stroke, Hiccup, Toothache, Stomachache, | Grinding, Decoction | Eaten, Body massage, Drinking, Bathing or steam bathing | Lemon, Ginger | None |
| Gyeene Kankan | *Allium sativum*L. | Liliaceae | C, F | Cough, Candidiasis, Stroke, Arthritis | Eaten raw, Grinding | Eaten, Drinking | Honey, Food | None |
| Alovera | *Aloe cf. tenuifolia*Lam. | Asphodelaceae | L | Typhoid fever, Diabetes, Skin disease | Decoction, Infusion | Drinking, Body massage | Fever grass | Itching skin |
| Nsoesoe/ Nkaseenkasee | *Alternanthera pungens*Kunth | Amaranthaceae | L, WP, SB | Asthma, Ulcer, Diarrhoea, Dysentery, Aseram, Delayed child walk | Grinding, Crushing, Decoction | Drinking, Inhalation, Bathing or Steam bathing, Ear or eye drop, | Ginger, Pepper, Nantwibini | Bitterness taste, Frequent running |
| ***Appendix Table 2 Continued*** | | | | | | | | |
| Nantwibini | *Amaranthus spinosus*L. | Amaranthaceae | WP, L | Dewormer, Malaria, Easy baby delivery, Delayed child walk | Decoction, Crushing | Drinking, Bathing or steam bathing | Esere, bark of a pineapple fruit, hot water | None |
| Aborobe | *Ananas comosus*(L.) Merr. | Bromeliaceae | R, L, F | Jaundice, Phlegm, Itching ear, Fever | Decoction, Grinding, Crushing | Drinking, Ear or eye drop | Neem leaf, Fever grass, Food | Allergic reaction |
| Aparagus dua | *Asparagus africanus* L. | Asparagaceae | R | Wound | Grinding | Body massage | None | None |
| Odwene/ Aduma | *Baphia nitida* Lodd. | Fabaceae | L, SB | Umbilical cord infections, Waist pain | Decoction | Drinking | None | None |
| Dwirentwi/ Gyinantwi | *Bidens pilosa*L. | Asteraceae | L, WP | Anaemia, Hypertension, Itching ear, Malaria, Aseram | Decoction, Grinding | Drinking, Ear or eye drop | Ginger | Hungry |
| Nkokodwe | *Boerhavia diffusa* L. | Nyctaginaceae | R | Gonorrhoea | Decoction | Drinking | None | Sleeping |
| Taa meawu | *Brachyachne obtusiflora*(Benth.) C.E. Hubb. | Poaceae | L | Delayed child walk, Fracture | Grinding, Infusion | Body massage | Fam wisa | None |
| Kwaebese | *Bryophyllum pinnatum*(Lam.) Oken | Crassulaceae | L, WP | Abdominal pains, Stroke, Stomach pains | Decoction, Eaten raw | Drinking, Eaten, Bathing or steam bathing | Alcohol, Salt | Sleeping, Nausea |
| Brofere | *Carapa procera* DC. | Meliaceae | SB | Chest pain | Decoction | Drinking | Alcohol | Nausea |
| Akyeampong | *Chromolaena odorata*(L.) R.M.King and H.Rob. | Compositeae | L | Fatigue, Typhoid, Wound, Stops bleeding | Decoction, Infusion, Grinding, Crushing | Inhalation, Body massage, Drinking | Spit, Water, Salt | Sharp pains, Sneezing |
|  |  |  |  |  |  |  |  |  |
|  |  |  |  |  |  |  |  |  |
| ***Appendix Table 2 Continued*** | | | | | | | | |
| Hwiremoo | *Combretum smeathmannii* G. Don. | Combretaceae | L | Easy baby delivery, Migraine | Crushing, Grinding, Decoction | Drinking | Cloves, Ginger | None |
| Ti- ahaban | *Cymbopogon citratus* (DC.) Stapf | Poaceae | L | Skin disorders, Fever, Malaria, Arthritis, | Decoction | Drinking | Dry pawpaw leaves, Beverage, Neem leaf, Lemon juice | None |
| Ahomakyem | *Dalbergia saxatilis*Hook.f. | Fabaceae | SB, ST, S, WP | Piles, Goitre, Fertility | Decoction, Infusion, Crushing | Bathing or steam bathing, Drinking, Body massage | Ginger, Wedieaba, Egg | Weakness, Running stomach |
| Ntum | *Eclipta alba*Hassk*.* | Asteraceae | L | Catarrh, Aseram, Malaria, Fever | Grinding, Infusion | Ear or eye drop, Drinking | None | Sneezing |
| Kakaweadwe | *Euphorbia hirta*L. | Euphorbiaceae | L | Wounds, Ulcer | Grinding | Body massage, Inhalation | Ginger, Soro wisa | Allergic reactions |
| Asaawa | *Gossypium hirsutum*L. | Malvaceae | L, S, R | Ulcer, Wound, Blood tonic, Vomiting, Abortion, Swellings, Headache | Decoction, Grinding | Drinking, Body massage | None | None |
| Kagya | *Griffonia simplicifolia* (DC.) Baill. | Fabaceae | S | Stomach ache, Ulcer | Decoction, Eaten raw | Drinking, Eaten | None | None |
| Akokotuatu/ Akomfemtikoro | *Heliotropium indicum*L. | Boraginaceae | L | Headache, Anaemia | Decoction, Crushing, Infusion, Grinding | Ear or eye drop, Drinking, Body massage | Shea butter, Solanum torum, Honey | Sneezing |
| Sesame | *Holarrhena floribunda* (G.Don.) Dur.and Schinz | Apocynaceae | S, B | Skin disorder, Different eye colour | Decoction | Drinking | None | Bloat |
| ***Appendix Table 2 Continued*** | | | | | | | | |
| Nunum nini | *Hoslundia opposita*Vahl. | Lamiaceae | L, S | Malaria | Decoction | Drinking | Neem leaf | None |
| Nkradedua | *Jatropha curcas*L. | Euphorbiaceae | R, L, F | Stomach ache, Hernia | Decoction | Ear or eye drop, Drinking | None | Running stomach |
| Afama | *Justicia flava*Vahl. | Acanthaceae | L | Swollen joint, Diarrhoea, Piles, Malaria | Decoction, Grinding | Body massage, Drinking, Eaten | Garlic, Boiling water, Neem, Ogyama | Running stomach, Bitterness |
| Egorɔ | *Kalanchoe integra*Kuntze. | Crassulaceae | L, WP | Phlegm, Stroke, Aseram, Cold | Decoction, Grinding, Crushing | Bathing or steam bathing, Drinking, Body massage | Cloves, Teak, Emire, Salt | None |
| Ananse dokono | *Lantana camara* L. | Verbenaceae | SB | Diarrhoea | Grinding | Body massage | Water | None |
| Bankye | *Manihot esculenta*Crantz | Euphorbiaceae | L, S | Stomachache, Stroke, Snake bite, Blood clotting, Constipation | Grinding, Crushing, Eaten raw | Body massage, Eaten, Drinking | Salt, Ngo ne nkyene, Food | Allergic reactions, Severe pain |
| Odubrafo | *Mareya micrantha* (Benth.) Müll.Arg. | Euphorbiaceae | L | Epilepsy, Waist pain, Stomachache | Decoction, Infusion, Grinding | Inhalation, Body massage, Drinking | Ginger, Pepper, Lime | None |
| Mfofo | *Melanthera scandens* Schu, Nach and Thonn | Asteraceae | R, L | Wounds | Decoction, Grinding | Body massage, Drinking | Salt, Shea butter | None |
| Militia | *Millettia ferruginea* (Hochst) Baker | Fabaceae | F | Skin infection | Grinding | Body massage | Shea butter | None |
| Mumuanka | *Mimosa pudica*L. | Fabaceae | L | Wounds | Grinding | Body massage | Salt | None |
|  |  |  |  |  |  |  |  |  |
|  |  |  |  |  |  |  |  |  |
|  |  |  |  |  |  |  |  |  |
|  |  |  |  |  |  |  |  |  |
|  |  |  |  |  |  |  |  |  |
| ***Appendix Table 2 Continued*** | | | | | | | | |
| Nyanya | *Momordica charantia*L. | Cucurbitaceae | L, R, B | Abdominal pains, fever, ringworm, Aseram, Measles, Gonorrhoea, Snakebite, Headache, Pot belly, Malaria, Diabetes | Grinding, Crushing, Decoction, Infusion | Bathing or steam bathing, Drinking, Body massage, Eye/ Ear drop | Soro wisa and fam wisa, Ginger, Alcohol, Peppercorn, Palm wine, Mahogany, Charcoal, Kaolin | Frequent urine, Running stomach, Vomiting, Bitter mouth, Allergic reaction |
| Dunsinkro | *Mormodica foetida* Schumach | Cucurbitaceae | R | Ear problem | Crushing | Ear or eye drop | None | None |
| Brodeε | *Musa paradisiaca*L. | Musaceae | R, L | Delayed child walk, Wounds, Headache | Crushing, Grinding | Bathing or steam bathing, Body massage, Eye/ Ear drop | None | None |
| Damerama | *Mussaenda erythrophylla* Schumach. and Thonn. | Rubiaceae | L | Severe cough, Heart disease | Decoction | Drinking | Oyaw, Lime | None |
| Aya | *Nephrolepis biserrata* (Sw.) Schott | Nephrolepidaceae | L | Blood tonic, Toothache | Infusion, Decoction, Crushing | Ear or eye drop, Eaten, Drinking | Mahogany, Alcohol, Salt | Headache |
| Bonto | *Nicotiana tabacum* Linnaeus | Solanaceae | L, R | Stomach pain, Headache | Grinding, Eaten raw | Bathing or steam bathing, Inhalation | Lemon | Collapse if not eaten, addiction |
| Nunum | *Ocimum gratissimum*L. | Lamiaceae | L, S | Stomach disorder, Malaria, Itchy ear, Phlegm, Convulsion | Grinding, Infusion, Decoction, Crushing | Bathing or steam bathing, Drinking, Eye/ Ear drop | Soro wisa, Pepper, Ginger, Lime, Dried Mango leaves | Bitter mouth, Allergic reactions |
| Duawusa | *Pachypodanthium staudtii* Engl. and Diels | Annonaceae | SB | Abdominal pains | Decoction | Drinking | None | None |
|  |  |  |  |  |  |  |  |  |
| ***Appendix Table 2 Continued*** | | | | | | | | |
| Abakamo | *Parquetina nigrescens* (Afzel). Bullock | Asclepiadaceae | L,SB | Piles, Poison antidote, Family planning, Fertility | Grinding | Body massage, Drinking | Shea butter | Slight pains |
| Toa ntini | *Paullinia pinnata*L. | Sapindaceae | R, L | HIV, Wounds Miscarriage, Snakebite, Profuse cough, Erectile dysfunction | Grinding, Eaten raw, Infusion, Decoction, Crushing | Body massage, Drinking, Eaten, Bathing or steam bathing | Ginger, Alcohol, Shea butter | Sleeping |
| Awobe | *Phyllanthus muellerianus* (Kuntze.) Exell. | Euphorbiaceae | L | Wounds | Infusion, Grinding | Body massage | None | Allergic reaction |
| Bowomaguwakyi/ Awommaguwakyi | *Phyllanthus urinaria*L. | Euphorbiaceae | WP, L | Cancer, Sore throat, High blood pressure | Decoction, Eaten raw, Grinding | Drinking, Eaten | Ginger, Honey, Salt, Coconut | Allergic reaction |
| Mayaabea | *Pteridium esculentum* Lucidcentral | Dennstaedtiaceae | L | Menstrual pains | Decoction | Drinking | None | None |
| Aposompo | *Pupalia lappacea* (L.) A Juss | Amaranthaceae | L | Boils, Swollen finger, Rashes | Crushing, Infusion | Ear or eye drop, Body massage | Palm oil, ashes | Allergic reactions |
| Adedenkruma | *Ricinus communis* L. | Euphorbiaceae | L | Measles, Hiccups | Decoction | Drinking | None | None |
| Ahwedeε | *Saccharum officinarum*L. | Poaceae | L | Malaria | Decoction | Drinking | Bamboo, Teak, Fever leaves | None |
| Sempe | *Senna alata*(L.) Roxb. | Fabaceae | L | Dandruff, Eczema, Skin rashes, Stomachache | Grinding, Decoction | Body massage, Drinking | Wediaba, Weed seed, Ashes, Prekese, Nantwibini | None |
| Sempe | *Senna alata*(L.) Roxb. | Fabaceae | L | Dandruff, Eczema, Skin rashes, Stomachache | Grinding, Decoction | Body massage, Drinking | Wediaba, Weed seed, Ashes, Prekese, Nantwibini | None |
| ***Appendix Table 2 Continued*** | | | | | | | | |
| Nkwadaa brodee | *Senna occidentalis*(L.) Link | Fabaceae | L, S, R | Severe vomiting, Diabetes, Gonorrhoea, Catarrh, Malaria | Grinding, Decoction | Ear/ Eye drop, Bathing or Steam bathing, Drinking | Neem leaves, Fam wisa | Bitter taste, Headache, Vomiting |
| Mmofrabrode | *Sesamum indicum*L. | Pedaliaceae | B | Blue eye sight, Cough | Decoction, Grinding, Eaten raw | Drinking, Eaten | Honey, Soft drink | None |
| Sesame | *Sida acuta* Burm. f. | Malvaceae | L, R | Pregnancy care, Cough, Sore throat | Grinding, Crushing | Bathing or steam bathing, Drinking | Prekese | Running stomach |
| Pepediewuo | *Solanum erianthum*D. Don | Solanaceae | L | Wounds, Piles | Grinding | Ear or eye drop, Body massage | None | None |
| Ntoose, Nsusuwa, Asamantrowa | *Solanum lycopersicum*L. | Solanaceae | F, L | Scorpion bite antidote, Wounds, Measles, Convulsion | Crushing, Grinding, Infusion | Body massage | None | None |
| Osisiriw/ Akuakua nisuo | *Solanum torvum*Sw. | Solanaceae | F, L | Blood tonic, Cough, Headache | Decoction, Grinding | Drinking | Tomatoes paste, Malt, Prekese | |
| Aduro kokoo/ Kramankote | *Sphenocentrum jollyanum*Pierre | Menispermaceae | R, WP | Erectile dysfunction, Low sperm count | Eaten raw, Decoction | Eaten | Alcohol, Water | Sleeping, Running stomach |
| Wawa bima | *Tapinanthus bangwenis* (Engl.and K. Krause) Danser | Loranthaceae | L, WP | Aseram, Sickle cell, Fertility, Pregnancy care, Fever | Decoction, Crushing | Bathing or steam bathing, Drinking | Palmnut, Smoked fish, dry Mango leaves | More urinating |
| Nkrangyedua | *Taraxacum officinale* F.H.Wigg. | Asteraceae | L, R | Blood tonic, Fever, Ulcer | Decoction, Grinding, Eaten raw | Drinking, Eaten | Pear leaves, Teak leaves, Malt, Food, Milk | None |
|  |  |  |  |  |  |  |  |  |
|  |  |  |  |  |  |  |  |  |
| ***Appendix Table 2 Continued*** | | | | | | | | |
| Awonwone | *Vernonia amygdalina*Delile | Asteraceae | L, R, WP | Aseram, High blood pressure, Malaria, Diarrhoea, Typhoid, Fever | Crushing, Decoction, Eaten raw, Infusion | Body massage, Drinking, Eaten | Kwekwenisuo, Pepper, Lemon, Cloves, Dandelion | Bitter taste |
| Mankani | *Xanthosoma mafaffa*Schott | Araceae | L | Snakebite antidote, Wounds, Blood tonic, Toothache | Crushing, Decoction, Grinding | Body massage, Drinking, Eaten | Prekese, Prekese, Salt | None |
| Aburoo | *Zea mays* L. | Poaceae | R, C | Constipation, Anal sore | Grinding | Body massage | None | Allergic reactions |
| Akakaduro | *Zingiber officinale*Roscoe | Zingiberaceae | F, B, RH | Sore throat, Wounds, Cough, Diarrhoea, Swollen fingers | Crushing, Decoction, Infusion, Grinding, Eaten raw | Eaten, Drinking, Inhalation, Body massage | Lemon, Garlic, Honey, Sugar | Allergic reactions, Bitter mouth |
| Awapuhi | *Zingiber zerumbet* L.Sm | Zingiberaceae | B | Syphilis | Decoction | Drinking | Alcohol | Dizziness |

MOP is method of preparation, MOA is method of administration, PPU is plant part used, B is bulb, SB is stem bark, S is seeds, RH is rhizome, F is fruit, C is cob, L is leaves, WP is whole plant, R is roots, and ST is small twigs.
