# Supplementary material for: Ethnobotanical Study of Medicinal Shrubs and Herbs Used by Forest-Fringe Communities of Ghana
Source: Scientifica (Cairo). 2025 May 19;2025:1362301. doi: 10.1155/sci5/1362301 (PMC12105890; doi:10.1155/sci5/1362301)
Supplement: Supporting Information 3 — Appendix Table 3: Informant agreement ratio values by categories for treating various diseases in the fringe communities of Asukese and Amama Shelterbelt Forest Reserves. [file 1362301.f3.docx]

**Appendix Table 3: Informant agreement ratio values by categories for treating various diseases and other health issues in the fringe communities of Asukese and Amama Shelterbelt Forest Reserves.**

| **Disease category** | **List of plant species used and number of uses** | **Number of taxa (Nt)** | **Number of mentions (Nur)** | **IAR** |
| --- | --- | --- | --- | --- |
| Analgestic, antiseptic and anti-inflamatory | *Paullinia pinnata L. (8), Mareya micrantha (Benth.) Müll.Arg. (5), Alternanthera pungens Kunth (2), Baphia nitida Lodd. (1), Justicia flava Vahl. (1), Nicotiana tabacum Linnaeus (1), Ocimum gratissimum L. (1)* | 7 | 19 | 0.667 |
| Anti-venom, anti-sting and repellants | *Momordica charantia L. (6), Paullinia pinnata L. (3), Xanthosoma mafaffa Schott (3), Manihot esculenta Crantz (2), Parquetina nigrescens (Afzel). Bullock (1), Solanum lycopersicum L. (1),* | 6 | 16 | 0.667 |
| Circulatory disorders | *Vernonia amygdalina Delile (13), Chromolaena odorata (L.) R.M.King and H.Rob. (6), Cymbopogon citratus (DC.) Stapf (6), Ananas comosus (L.) Merr. (4), Ocimum gratissimum L. (4), Saccharum officinarum L. (4), Bidens pilosa L. (3), Eclipta alba Hassk. (3), Heliotropium indicum L. (3), Justicia flava Vahl. (3), Hoslundia opposita Vahl. (2), Senna occidentalis (L.) Link (2), Xanthosoma mafaffa Schott (2), Alchornea cordifolia (Schumach. and Thonn.) Müll.Arg. (1), Amaranthus spinosus L. (1), Manihot esculenta Crantz (1), Momordica charantia L. (1), Phyllanthus urinaria L. (1)* | 18 | 60 | 0.712 |
| Dental problems | *Nephrolepis biserrata (Sw.) Schott (4), Alchornea cordifolia (Schumach. and Thonn.) Müll.Arg. (1), Senna occidentalis (L.) Link (1), Xanthosoma mafaffa Schott (1)* | 4 | 7 | 0.500 |
| Developmental delay | *Musa paradisiaca L. (3), Alternanthera pungens Kunth (2), Amaranthus spinosus L. (1), Brachyachne obtusiflora (Benth.) C.E. Hubb. (1)* | 4 | 7 | 0.500 |
| Digestive disorders | *Alternanthera pungens Kunth (7), Vernonia amygdalina Delile (7), Bryophyllum pinnatum (Lam.) Oken (6), Griffonia simplicifolia (DC.) Baill. (4), Justicia flava Vahl. (4), Ocimum gratissimum L. (4), Taraxacum officinale F.H.Wigg. (4), Zingiber officinale Roscoe (4), Momordica charantia L. (3), Paullinia pinnata L. (3), Alchornea cordifolia (Schumach. and Thonn.) Müll.Arg. (2), Manihot esculenta Crantz (2), Senna occidentalis (L.) Link (2), Zea mays (2), Ageratum conyzoides L. (1), Allium sativum L. (1), Amaranthus spinosus L. (1), Chromolaena odorata (L.) R.M.King and H.Rob. (1), Euphorbia hirta L. (1), Gossypium hirsutum L. (1), Jatropha curcas L. (1), Lantana camara L. (1),*  *Mareya micrantha (Benth.) Müll.Arg. (1), Nicotiana tabacum Linnaeus (1), Pachypodanthium staudtii (1), Tapinanthus bangwenis (Engl.and K. Krause) Danser (1)* | 26 | 66 | 0.615 |
| ***Appendix Table 4 Continued*** | | | | |
| Ear, Eye, Nose and Throat problems | *Ocimum gratissimum L. (7), Ananas comosus (L.) Merr. (5), Sida acuta Burm. f. (5), Bidens pilosa L. (3), Sesamum indicum L. (3), Zingiber officinale Roscoe (3), Ageratum conyzoides L. (2), Holarrhena floribunda (G.Don.) Dur.and Schinz (1), Mormodica foetida Schumach (1), Phyllanthus urinaria L. (1), Pupalia lappacea (L.) A Juss (1)* | 11 | 32 | 0.677 |
| Endocrine disorders | *Aloe cf. tenuifolia Lam. (7), Momordica charantia L. (1), Senna occidentalis (L.) Link (1)* | 3 | 9 | 0.750 |
| Integumentary disorders | *Senna alata (L.) Roxb. (20), Acacia pennata Willd. (9), Chromolaena odorata (L.) R.M.King and H.Rob. (8), Pupalia lappacea (L.) A Juss (8), Solanum erianthum D. Don (4), Melanthera scandens Schu, Nach and Thonn (4), Musa paradisiaca L. (3), Paullinia pinnata L. (3), Phyllanthus muellerianus (Kuntze.) Exell. (3), Gossypium hirsutum L. (3), Xanthosoma mafaffa Schott (2), Abelmoschus esculentus Moench. (1), Alchornea cordifolia (Schumach. and Thonn.) Müll.Arg. (1), Aloe cf. tenuifolia Lam. (1), Asparagus africanus L. (1), Cymbopogon citratus (DC.) Stapf (1), Euphorbia hirta L. (1), Holarrhena floribunda (G.Don.) Dur.and Schinz (1), Millettia ferruginea (1), Mimosa pudica L. (1), Momordica charantia L. (1), Solanum lycopersicum L. (1), Zingiber officinale Roscoe (1)* | 23 | 79 | 0.718 |
| Internal medical problems | *Vernonia amygdalina Delile (8), Cymbopogon citratus (DC.) Stapf (7), Aloe cf. tenuifolia Lam. (6), Momordica charantia L. (5), Ananas comosus (L.) Merr. (4), Tapinanthus bangwenis (Engl.and K. Krause) Danser (4), Taraxacum officinale F.H.Wigg. (4), Chromolaena odorata (L.) R.M.King and H.Rob. (3), Combretum smeathmannii G. Don. (3), Heliotropium indicum L. (3),Allium sativum L. (2), Acacia pennata Willd. (2), Amaranthus spinosus L. (2), Senna alata (L.) Roxb. (2), Zea mays (2), Eclipta alba Hassk. (1), Gossypium hirsutum L. (1), Abrus precatorius L. (1), Ageratum conyzoides L. (1), Alchornea cordifolia (Schumach. and Thonn.) Müll.Arg. (1), Justicia flava Vahl. (1), Musa paradisiaca L. (1), Mussaenda erythrophylla Schumach. and Thonn. (1), Ocimum gratissimum L. (1), Solanum torvum Sw. (1)* | 25 | 67 | 0.636 |
| Neurological disorders | *Abrus precatorius L. (8), Ocimum gratissimum L. (5), Paullinia pinnata L. (3), Aframomum melegueta K.Schum. (2), Kalanchoe integra Kuntze. (2), Manihot esculenta Crantz (2), Alchornea cordifolia (Schumach. and Thonn.) Müll.Arg. (1), Allium sativum L. (1), Bryophyllum pinnatum (Lam.) Oken (1), Mareya micrantha (Benth.) Müll.Arg. (1), Solanum lycopersicum L. (1)* | 11 | 27 | 0.615 |
| Nutritional problems and tonic | *Taraxacum officinale F.H.Wigg. (7), Gossypium hirsutum L. (5), Nephrolepis biserrata (Sw.) Schott (5), Momordica charantia L. (3), Solanum torvum Sw. (3), Xanthosoma mafaffa Schott (3), Alchornea cordifolia (Schumach. and Thonn.) Müll.Arg. (1),* | 7 | 27 | 0.769 |
|  |  |  |  |  |
| ***Appendix Table 4 Continued*** | | | | |
| Others | *Chromolaena odorata (L.) R.M.King and H.Rob. (6), Momordica charantia L. (6), Vernonia amygdalina Delile (5), Eclipta alba Hassk. (4), Tapinanthus bangwenis (Engl.and K. Krause) Danser (4), Kalanchoe integra Kuntze. (4), Zingiber officinale Roscoe (3), Alternanthera pungens Kunth (3), Dalbergia saxatilis Hook.f. (2), Paullinia pinnata L. (2), Phyllanthus urinaria L. (1), Alchornea cordifolia (Schumach. and Thonn.) Müll.Arg. (1), Bidens pilosa L. (1), Senna occidentalis (L.) Link (1), Gossypium hirsutum L. (1), Pupalia lappacea (L.) A Juss (1), Senna occidentalis (L.) Link (1)* | 17 | 46 | 0.644 |
| Reprodutive problems | *Paullinia pinnata L. (17), Dalbergia saxatilis Hook.f. (15), Momordica charantia L. (8), Solanum erianthum D. Don (6), Sphenocentrum jollyanum Pierre (6), Sida acuta Burm. f. (5), Parquetina nigrescens (Afzel). Bullock (4), Combretum smeathmannii G. Don. (3), Justicia flava Vahl. (3), Abelmoschus esculentus Moench. (2), Ageratum conyzoides L. (2), Allium sativum L. (2), Pteridium esculentum Lucidcentral (2), Tapinanthus bangwenis (Engl.and K. Krause) Danser (2), Alchornea cordifolia (Schumach. and Thonn.) Müll.Arg. (1), Amaranthus spinosus L. (1), Baphia nitida Lodd. (1), Boerhavia diffusa (1), Gossypium hirsutum L. (1), Jatropha curcas L. (1), Senna occidentalis (L.) Link (1), Zingiber zerumbet L. Sm (1)* | 22 | 85 | 0.750 |
| Respiratory disorders | *Ocimum gratissimum L. (8), Zingiber officinale Roscoe (7), Eclipta alba Hassk. (6), Mussaenda erythrophylla Schumach. and Thonn. (6), Allium sativum L. (5), Paullinia pinnata L. (4), Kalanchoe integra Kuntze. (3), Momordica charantia L. (3), Sida acuta Burm. f. (3), Alternanthera pungens Kunt (2), Ricinus communis L. (2), Solanum lycopersicum L. (2), Solanum torvum Sw. (2), Ananas comosus (L.) Merr. (1), Carapa procera DC. (1), Chromolaena odorata (L.) R.M.King and H.Rob. (1), Senna occidentalis (L.) Link (1), Sesamum indicum L. (1)* | 18 | 58 | 0.702 |
| Skeletal problems | *Paullinia pinnata L. (4), Ananas comosus (L.) Merr. (3), Justicia flava Vahl. (3), Alchornea cordifolia (Schumach. and Thonn.) Müll.Arg. (2), Brachyachne obtusiflora (Benth.) C.E. Hubb. (2), Allium sativum L. (1), Cymbopogon citratus (DC.) Stapf (1)* | 7 | 16 | 0.600 |

IAR = Informant Agreement Ratio
